# Ovarian Transcriptome Analysis of *Portunus trituberculatus* Provides Insights into Genes Expressed during Phase III and IV Development

**DOI:** 10.1371/journal.pone.0138862

**Published:** 2015-10-02

**Authors:** Yunxia Yang, Jiteng Wang, Tao Han, Tao Liu, Chunlin Wang, Jia Xiao, Changkao Mu, Ronghua Li, Fangping Yu, Huilai Shi

**Affiliations:** 1 Fisheries College of Zhejiang Ocean University, Zhoushan, China; 2 Annoroad Gene Technology Co., Ltd, Beijing, China; 3 Marine Science College of Ningbo University, Ningbo, China; 4 Department of Immunobiology, Jinan University, Guangzhou, China; 5 Marine Fisheries Research Institute of Zhejiang Province, Zhoushan, China; Shanghai Ocean University, CHINA

## Abstract

Enhancing the production of aquatic animals is crucial for fishery management and aquaculture applications. Ovaries are specialized tissues that play critical roles in producing oocytes and hormones. Significant biochemical changes take place during the sexual maturation of *Portunus trituberculatus*, but the genetics of this process has not been extensively studied. Transcriptome sequencing can be used to determine gene expression changes within specific periods. In the current study, we used transcriptome sequencing to produce a comprehensive transcript dataset for the ovarian development of *P*. *trituberculatus*. Approximately 100 million sequencing reads were generated, and 126,075 transcripts were assembled. Functional annotation of the obtained transcripts revealed important pathways in ovarian development, such as those involving the vitellogenin gene. Also, we performed deep sequencing of ovaries in phases III and IV of sexual maturation in *P*. *trituberculatus*. Differential analysis of gene expression identified 506 significantly differentially expressed genes, which belong to 20 pathway, transporters, development, transcription factors, metabolism of other amino acids, carbohydrate and lipid, solute carrier family members, and enzymes. Taken together, our study provides the first comprehensive transcriptomic resource for *P*. *trituberculatus* ovaries, which will strengthen understanding of the molecular mechanisms underlying the sexual maturation process and advance molecular nutritional studies of *P*. *trituberculatus*.

## Introduction

*Portunus trituberculatus* is an important commercial seafood found in the oceans around Korea, Japan, China, and Taiwan [1–3]. For its high nutritional value, market demand is increasing. However, production of *P*. *trituberculatus* has dramatically declined during the past decade due to overfishing and environmental deterioration [4], which has advanced the artificial culture of *P*. *trituberculatus* in China [5]. During culture of this crab, it has been difficult to maintain stable production due a lack of basic knowledge about its growth and maturation.

Ovaries are important tissues in reproduction and fulfill many pivotal functions, including oocyte production, hormone secretion, and fertilization. Some research has shown that ovarian development can be divided into six stages [6]. There are major morphological and histological differences in crab ovaries between phases III and IV. In phase III, ovaries are buff and orange, and the main cell type in the ovary is exogenous vitellogenic oocyte. This marks a period of rapid physical development for the ovaries. In phase IV, the ovaries are deep orange, with the main cell type being mature oocytes. In this phase, the egg developed rapidly and became maturity in the ovaries. In female *P*. *trituberculatus*, ovarian development usually starts after puberty molt and mating. Based on analysis of the yolk, the process of ovarian development can be divided into endogenous and exogenous vitellogenic stages. Both phases III and IV of ovarian development belong to the exogenous vitellogenic stage. From phase III to IV, ovaries undergo massive increases in volume and weight as body material storage increases, as well as gamete production [7]. To obtain a better understanding of *P*. *trituberculatus* reproduction, it is very important to explore the genetic basis of the ovary, with an emphasis on development and sexual maturation processes. Thus, there is an urgent need to investigate the mechanisms of *P*. *trituberculatus* growth and maturation under natural conditions and to develop novel means to enhance *P*. *trituberculatus* production artificially.

So far as genome sequence resources for crabs, it is still unavailable. This situation limits the ability to gain a molecular understanding of physiological processes in these crabs. The advent of next-generation sequencing technologies has fundamentally accelerated biological research by providing huge amounts of data in a short time at low cost. Transcriptome sequencing enables the production of high-throughput fragments of double-stranded cDNA and the rapid assembly of sequences for annotation. It facilitates gene discovery and broadens our understanding of gene networks, especially in non-model organisms with unknown genomes [8]. For example, this technology has successfully been used to discover genes involved in immune pathways in the hepatopancreas of the microbially-challenged mitten crab *Eriocheir sinensis* [9].

*P*. *trituberculatus* genomics remains a mostly unexplored area of research. Wang et al. sequenced the transcriptome about the hepatopancreas of *P*. *trituberculatus* using 454 high-throughput pyrosequencing method [10]. Although there are some information about the ovarian development, the main content is about hepatopancreas transcriptome To explore the mechanisms of molecular regulation during crab ovary maturation, we performed single-end RNA sequencing of *P*. *trituberculatus* ovaries at phases III and IV of development with biological replicates. The target of this study was to acquire the ovarian transcriptome of this crab as it applies to the study of molecular mechanisms underlying physiological and morphological changes during the phase III to phase IV transition in ovarian development using Illumina HiSeq sequencing. Following transcriptome sequencing and annotation, 126,075 contigs were assembled using Trinity software, which can reconstruct a large fraction of full-length transcripts [11]. This study represents the first exploration about the ovarian transcriptome of the *P*. *trituberculatus* with large-scale high-throughput sequencing. Our results may serve to guide further functional genomics studies, as well molecular nutritional studies of crustacean.

## Materials and Methods

### Ethics statement

Wild *P*. *trituberculatus* were obtained from the ocean surrounding Xiangshan, Zhejiang Province, China, in May, 2012. The sampling location was not privately-owned or protected, and field sampling did not involve protected species. All fishing activities were approved by the Zhejiang Province Ocean and Fisheries Bureau.

### RNA extraction and sequencing

Crabs were kept under laboratory conditions with 22°C temperature water and 28 g/L salinity. We chose six crabs for sequencing: three in phase III and three in phase IV of ovarian development. We dissected all six crabs and froze ovarian tissues with liquid nitrogen. Total RNA was extracted from the ovaries using TRIzol reagent (Invitrogen) following the manufacturer’s instructions and treated with DNase I. The concentration and quality of total RNA were determined using NanoDrop 2000 (Thermo Scientific). Total ovarian RNA from one crab in phase III and one in phase IV were balanced mixed for paired-end RNA sequencing; total ovarian RNA from the remaining four crabs was used for single-end RNA sequencing. Library construction and sequencing were performed on an Illumina HiSeq2000 sequencer according to the manufacturer’s specifications (Illumina).

### *De novo* assembly and functional annotation

Paired-end sequencing results (RQ12BJ00208) of mixed RNA from phases III and IV of ovarian development were used for *de novo* assembly. Raw reads were preprocessed by discarding reads with adaptors and low quality (quality scores <30) and then were assembled using Trinity software with default parameters [11, 12]. Transcripts shorter than 200 bp were removed for subsequent analysis.

Transcripts from the previous step were translated in all six possible open reading frames (ORFs). Proper translation was defined as the one that gave the longest amino acid sequence [13]. Using the hidden Markov model (HMM), we filtered and obtained optimal amino acid sequences. The functional annotations of predicted amino acid sequences were performed using Trinotate (http://trinotate.sourceforge.net/) by searching against the Uniprot Knowledgebase and Swiss-Prot. Meanwhile, we ran HMMER, signalIP, and TMHMM in Trinotate to identify protein domains and predict signal peptides and transmembrane regions, respectively.

Afterwards, Gene Ontology (GO) annotations of transcripts were obtained by searching from the non-redundant database using Blast2GO [14] with an E-value cutoff of 10^−5^. GO functional classifications were performed with WeGO software [15]. Kyoto Encyclopedia of Genes and Genomes (KEGG) pathways were used to assemble transcripts with the KEGG Automatic Annotation Server (http://www.genome.jp/kegg/kaas/).

### Assembly evaluation

To compare transcripts to those of a related species and known sequences of *P*. *trituberculatus*, unigenes of *Eriocheir sinensis* were downloaded from the National Center for Biotechnology Information (NCBI; Accession Number: KA660105-KA728674); expressed sequence tags (ESTs) and nucleotide sequences of *P*. *trituberculatus* were also extracted from the NCBI nucleotide database. All sequences were compared with our assembled transcriptomic sequences using Blat [16] with default parameters to evaluate the quality of the assembly.

### Identification of differentially expressed genes (DEGs)

Single-end RNA sequencing results from two crabs in phase III (RQ12BJ00204 and RQ12BJ00206) and two in phase IV (RQ12BJ00210 and RQ12BJ00212) were mapped to the assembled transcriptome using bowtie-1.0.1 [17]. The mapped read counts for each transcript were normalized by RPKM [18], which is able to eliminate the influence of different gene lengths and sequencing levels using RSEM [19]. Therefore, the calculated gene expression can be directly used for comparing differences in gene expression between samples. Then, we calculated differences in the abundance of expression of each gene on each transcript between two samples using DESeq [20] with a FDR <0.05 and an absolute value of fold-change ≥2. Biological repetitive analysis was carried out to evaluate the quality of sequence data.

### qRT-PCR of differentially expressed genes in phase III and phase IV of ovarian development

To validate the quantitative data of DGE libraries, we have quantified the expression levels of single gene. The nucleotide sequences of primers for single gene were designed following the sequences using Illumina sequencing (Table 1). Complementary DNA (cDNA) was synthesized from total RNAs, which obtained from ovaries of crabs in the two phases, and were also used in the single-end sequencing above. The qPCR amplifications were performed in FTC-2000 (Shanghai) in a final volume of 50μl containing 25μl of PCR buffer, 1μl of each primer, 0.5μl of SYBR Green I, 2μl of diluted cDNA, and 20.5μl of DEPC water. The PCR program was with an initial denaturation step of 10 min at 95°C, followed by an amplification of the target cDNA for 35 cycles, each cycle consisting of a denaturation at 95°C for 20s, annealing at 60°C for 30s and elongation at 72°C for 30s. The analysis was based on the Ct values of the PCR products. Results are shown as changes in relative expression normalized with β-actin using the 2^-(ΔΔCt)^ method described by Pfaffl [21].

**Table 1:** Nucleotide sequences of primers used for qPCR amplification.

Target gene	Forward (5′–3′)	Reverse (5′–3′)	length (bp)
β-actin	TCACACACTGTCCCCATCTACG	ACCACGCTCGGTCAGGATTTTC	114
Aspartate aminotransferase	AGCAGCGACCACCTGACG	CCTTCCACCCTCCTCTAAGTGA	183
Rsk1/2	CAAAAGGAACCCAGCGAACA	CCTGGTCGAAGTAGAAGGTGTCA	170
c59264_g1_i1	GGTAAGATGAAGCAGCGATGG	CCCAACCGTGCTCCTGTG	188
Genome polyprotein	CCGCAACAATCCAAAAGACC	AATGCCGCTATCGCAGTAAG	130
c96546_g1_i1	CCTAAGCCAAGCAGGATGTG	GTTGCTTCTGATGTAGTTTTCTGC	165
Granulins	GACGGCTCACTTGGGACTG	TATCGCTGTGCTTCTGGAGG	113

## Results

### *De novo* assembly of the transcriptome

**Fig 1:**
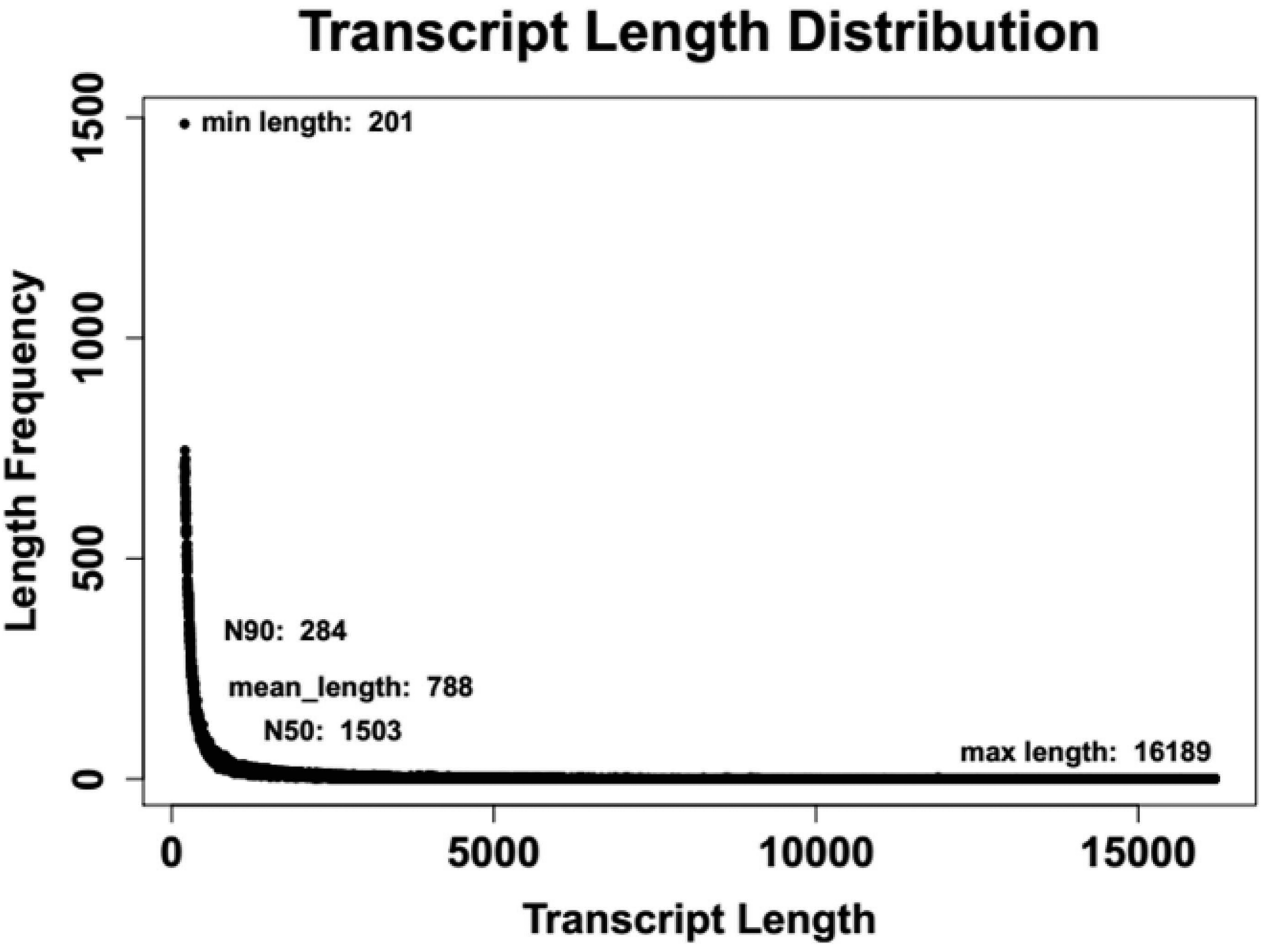
Sequence length distribution of transcripts assembled from Illumina reads.

Contigs (126,075) were generated from the library giving a total of ~100 million sequence reads (Table 2). There are 48,444 contigs with lengths up to 500 bp. The median size of the contigs (N_50_) was 1503 bp. The average size of the assembled contigs was 788 bp, and the lengths of contigs ranged from 200 bp to more than 16,000 bp (Fig 1). The Q30 bases rate, which is considered a benchmark for quality in next-generation sequencing, was up to 92.23%. All reads were deposited in the Short Read Archive (SRA) of the National Center for Biotechnology Information (NCBI) with the accession number SRX1081182.

**Table 2:** Summary of ovary transcriptome sequencing results of *Portunus trituberculatus*.

Assembly	ovary	ORF
Reads number	191,845,918	
Q30 bases rate (%)	92.23	
Total Contigs	126,075	36,343
Mean length (bp)	788	1,178
Median length (bp)	376	801
Max length (bp)	16,190	15,948
>500 bp	48,444	25,163
N_50_ value	1,503	1,653
GC content (%)	45.73	52.00

### Assembly validation

To further verify the quality of the transcriptome, we compared our assembled transcripts with ESTs and nucleotide sequences of *P*. *trituberculatus* from NCBI databases, as well as unigenes of *E*. *sinensis*. Using Blat searches, 21.13% of *P*. *trituberculatus* EST sequences were found to be aligned to the assembled transcripts with a sequence similarity greater than 50%. According to the same standard, 93.22% of nucleotide sequences were covered by our assembly. Besides the EST and nucleotide sequences of *P*. *trituberculatus*, we also performed a comparison between our assembly and the unigenes of *E*. *sinensis*. Sequences in our assembly had greater than 50% similarity with 29.87% of *E*. *sinensis* unigenes (Table 3).

**Table 3:** Comparison of *P*. *trituberculatus* ovarian transcriptome with annotations transcripts from NCBI.

		Similarity with *P*. *trituberculatus* ovary transcripts	
	Total	> 0.5 (*P*. *trituberculatus*)	> 0 (*P*. *trituberculatus*)
NCBI EST^1^	14,372	1,423 (3,163)	3,038 (27,956)
NCBI Nucleotide^2^	1,077	213 (1,115)	1,004 (13,652)
*E*. *sinensis* transcripts^3^	68,569	6,967 (16,437)	20,442 (61,019)

^1^ *P*. *trituberculatus* ESTs from the NCBI EST database

^2^ *P*. *trituberculatus* sequences from the NCBI nt database

^3^Transcriptome of Accessory Sex Gland and Testis in *E*. *sinensis*

### Functional annotation of the transcriptome

In our analysis, 36,343 contigs that were predicted to have ORFs were acquired, which represent 28.82% of the total assembled contigs. The lengths of the predicted ORFs range from 300–15,948 bp, with a median and mean size of 801 bp and 1178 bp, respectively (Table 2). Annotations of putative protein sequences translated from predicted ORFs were searched against various databases, such as NCBI, Swiss-Prot, and KEGG, using Trinotate and Blast. Overall, 29,296 (80.61%) putative proteins were annotated with at least one annotation. The statistics of annotation results are shown in S1 Table.

**Fig 2:**
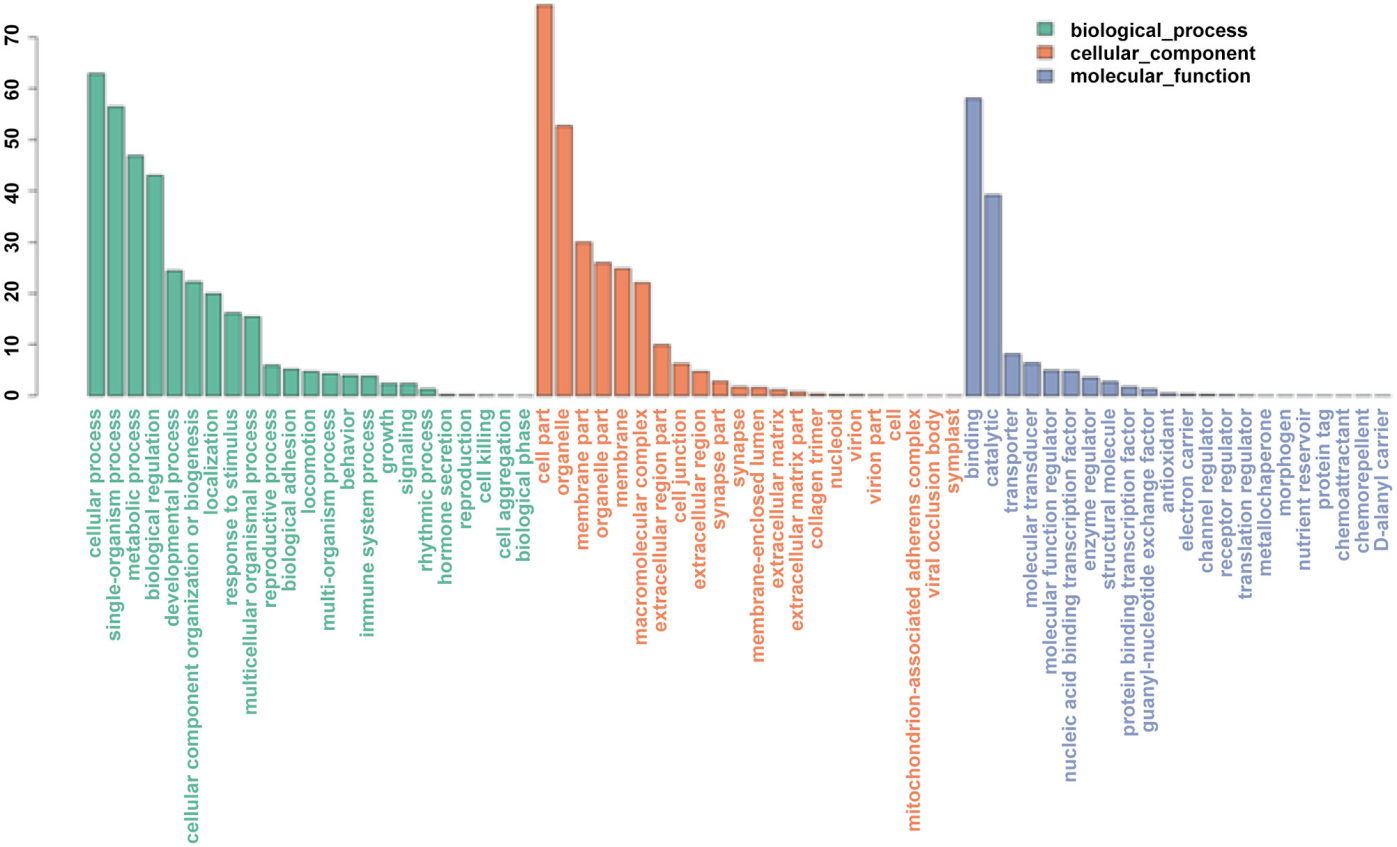
Gene Ontology classification of assembled gene s in the *P*. *trituberculatus* ovary transcriptome.

A total of 19,183 (52.78%) transcripts of *P*. *trituberculatus* were assigned for GO analysis using Blast2GO software. These transcripts were assigned with one or more GO terms, which fell into all three major GO categories, including cellular component, biological process, and molecular function (Fig 2). In the biological process category, cellular (70.15%) and metabolic processes (51.58%) were the most abundant GO terms. For molecular function, GO terms of binding (57.39%) and catalytic activity (38.81%) were highly represented. For cellular components, the most abundant categories were cell (82.22%) and cell part (82.22%).

**Fig 3:**
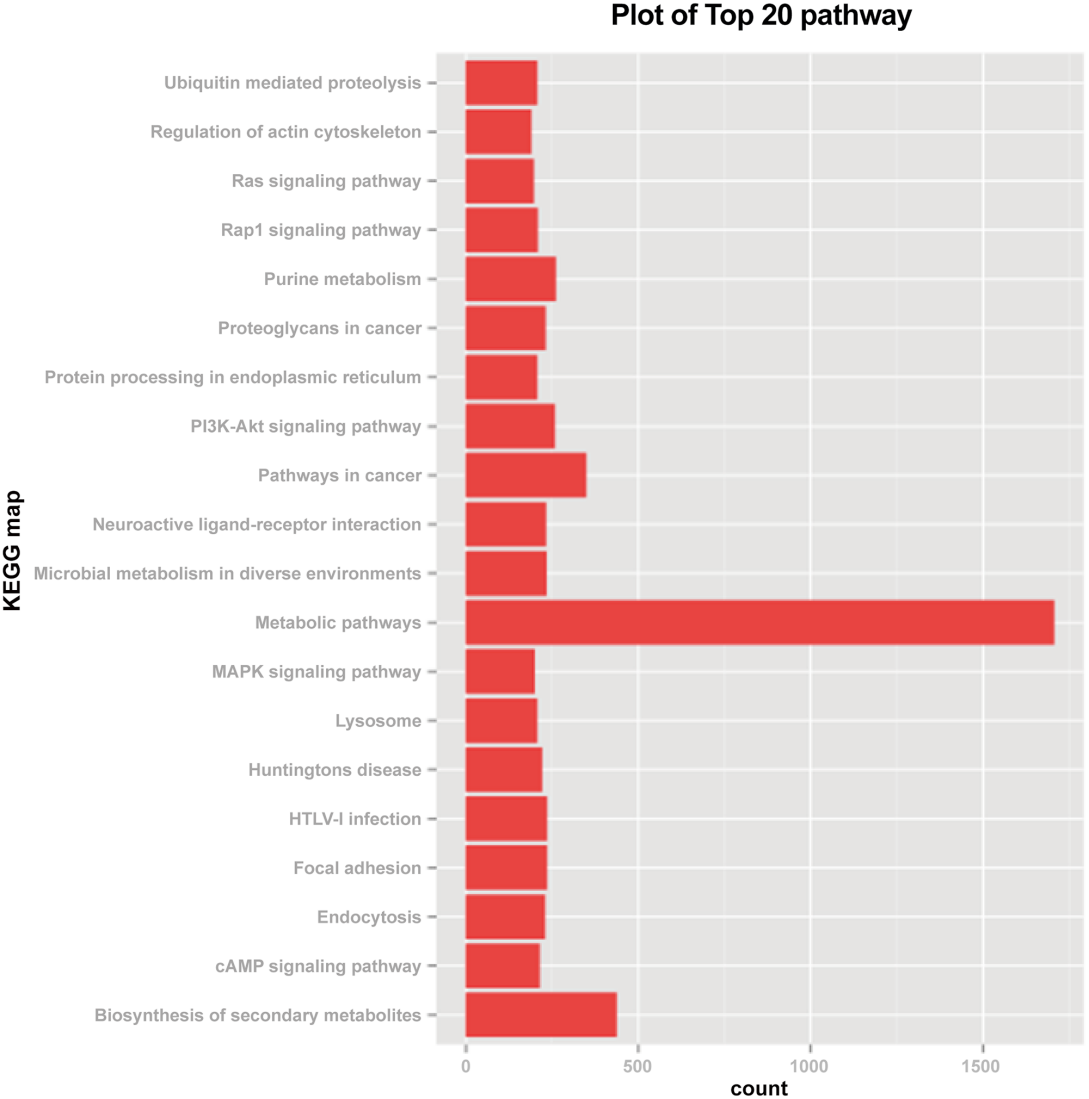
KEGG map in assemble result.

To further analyze the possible pathways involved in ovarian maturation, KEGG analysis was applied based on transcriptome results. We mapped 12,219 transcripts to 337 pathways (S2 Table) representing biological systems involved in the transition from phase III to IV in *P*. *trituberculatus* ovarian development. As shown in Fig 3, the most abundant pathways represented in the ovary are metabolic (1706 transcripts), biosynthetic pathways of secondary metabolites (437 transcripts), and purine metabolism (260 transcripts). These predicted pathways are likely to be of interest in future investigations by focusing on their functions in *P*. *trituberculatus*.

### DEGs between different phases of ovarian development

We performed single-end RNA sequencing for ovaries at phases III and IV of development with biological replicates. Approximately 20–30 million reads were generated for four ovarian sequencing libraries (phase III: RQ12BJ00204 and RQ12BJ00206; phase IV: RQ12BJ00210 and RQ12BJ00212; S3 Table). By mapping single-end RNA sequencing reads to transcripts assembled above, we obtained a more than 85% mapping rate for all libraries, which further implies a high quality of *de novo* assembly of transcripts. The expression of genes was measured for each library in RPKM. Clustering analysis based on the Pearson correlation of gene expression among samples showed that ovaries from the same phase have strong correlations distinct from samples from another phase (P < 2.2E-16 for each comparison, S1 Fig).

Totally, 506 genes were found to be significantly differentially expressed, including 482 upregulated and 24 downregulated genes in phase IV compared with phase III. To understand the functions of these DEGs, GO and KEGG analyses were performed. All DEGs were assigned to 1895 GO terms and 63 pathways through the KEGG database (S4 Table). Differentially expressed genes are mostly enriched in cell processes and biological regulation in the biological process category. In the pathway category, metabolic pathways encompassed the largest proportion which suggests strong nutritional metabolism during ovarian development of *P*. *trituberculatus* (S4 Table).

### qRT-PCR of differentially expressed genes in phase III and phase IV of ovary development

**Fig 4:**
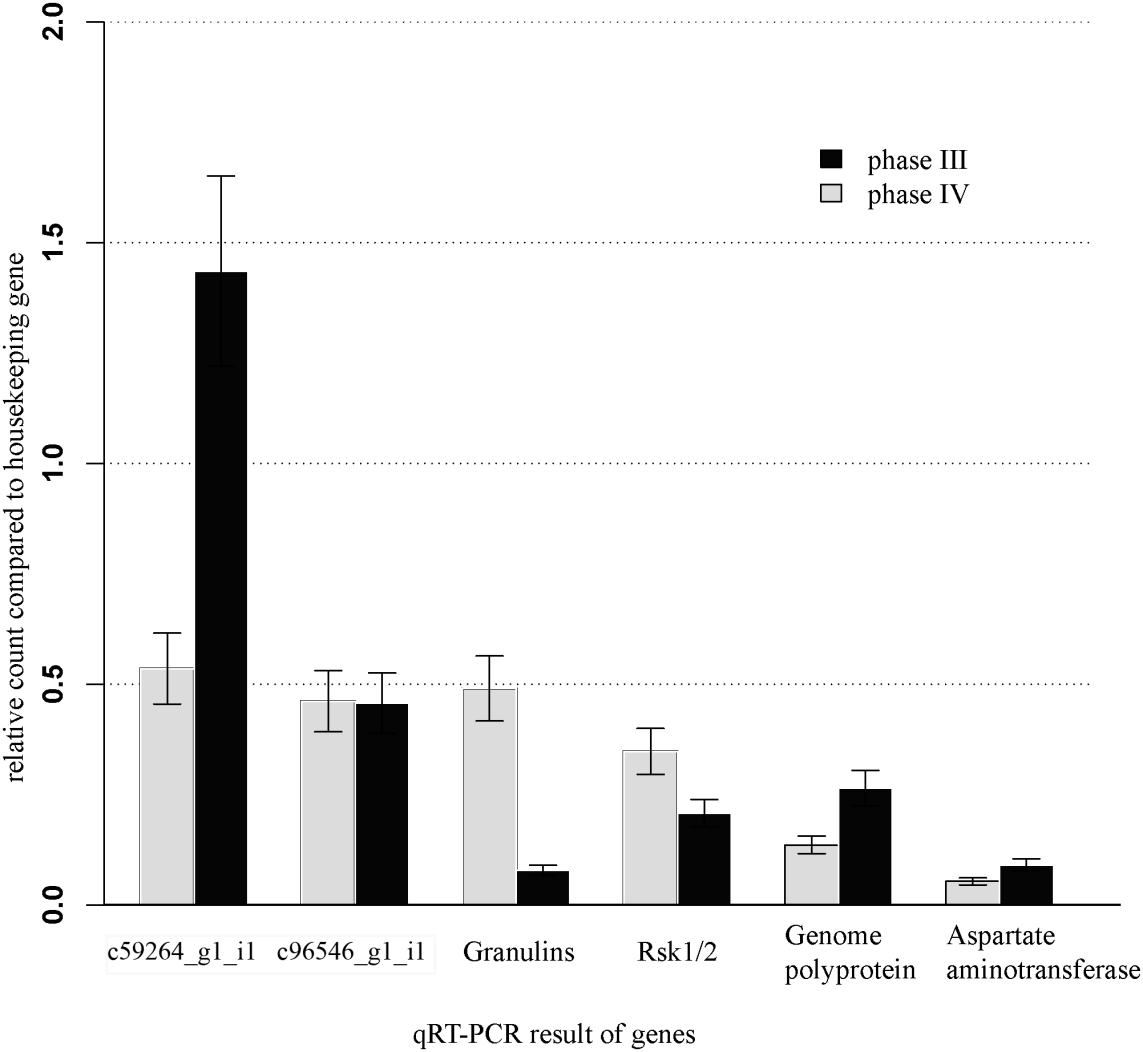
Quantitative expression of six genes were determined by qRT-PCR in two phases of ovary.

To validate the expression of DEGs, we further used qRT-PCR to examine expression levels of 6 genes in samples from phase III and phase IV of ovary development, respectively. As shown in Fig 4, expression of all genes can be detected, and the patterns of differential expression are consistent with the results from deep sequencing.

## Discussion

Ovarian development is an important and critical physiological process for crustacean reproduction. However, during artificial culture, crabs often have poor reproductive performance [1]. To identify the molecular signaling pathways related to crab ovarian development is helpful for understanding the molecular mechanism involved. Regarding molecular mechanisms of ovarian development, little is known about *P*. *trituberculatus* as much research has focused on *Callinectes sapidus* [22–24]. Wang *et al*. [10] has previously investigated the transcriptome of different hepatopancreas and ovarian stages of *P*. *trituberculatus*. In their research, a total of five cDNA libraries (i.e., growth, endogenous vitellogenic, and exogenous vitellogenic stage hepatopancreas; endogenous and exogenous vitellogenic stage ovaries) were constructed to sequence. But the information the about ovary stage was not enough, as only 50,062 reads were sequenced in the exogenous vitellogenic stage. In our study, nearly 100 million sequence reads were generated from the library, and 126,075 contigs were assembled. This result may give us more information about ovarian development in *P*. *trituberculatus*.

### Related processes and genes in ovarian development of *P*. *trituberculatus*

In the current study, the aim of our transcriptome sequencing project was to identify the genes involved in ovarian development. Using deep sequencing technology, we performed *de novo* assembly of the transcriptome of *P*. *trituberculatus* ovaries. According previous research, crustacean ovary development is controlled by hormones expressed in the ovaries [25]. 3-β-hydroxysteroid dehydrogenase, mandibular organ-inhibiting hormone, lutropin-choriogonadotropic hormone receptor, gonadotropin-releasing hormone receptor, follicle-stimulating hormone receptor, and estrogen related genes (estrogen sulfotransferase and estrogen receptor) were all found in our study (Table 4). These genes, which are part of ovarian steroidogenesis (map04913), are key genes for steroid hormone biosynthesis in crustaceans [26, 27]. The 3-β-hydroxysteroid dehydrogenase enzymatic system plays an important role in the biosynthesis of hormonal steroids in ovarian development by catalyzing the synthesis of progesterone from pregnenolone, 17-OH-progesterone from 17-OH-pregnenolone, and androstenedione from dehydroepiandrosterone. It has been shown that reproductive process in crustaceans is highly controlled and regulated by endocrine system [28]. Locally expressed steroids and hormones induce growth, differentiation, and maturation of follicular cells. Also, the level of hormone can provide useful information for judging the stage of ovarian development.

**Table 4:** Candidate genes involved into ovarian development of *P*. *trituberculatus*.

Name	Contig	Length	Pfam	Ident	E_value	Description
vitellogenin	c83640	1674	PF00057.13	39.27	5.00E-25	Vitellogenin receptor
	c94641	1801	PF00057.13	25.81	1.00E-07	Vitellogenin receptor
	c2459	1011	PF01347.17	48.01	5.00E-103	Vitellogenin
	c98213	4508	PF00094.20	42.69	0	Vitellogenin
	c95168	1410	PF00094.20	30.83	2.00E-70	Vitellogenin
	c95168	2814	.	21.61	4.00E-68	Vitellogenin
	c95168	1586	PF00094.20	29.57	5.00E-72	Vitellogenin
	c95168	1949	PF09172.6	38.22	1.00E-163	Vitellogenin
	c98213	3797	PF01347.17	42.69	0	Vitellogenin
	c91269	692	PF03762.12	40	3.00E-15	Vitelline membrane outer layer protein 1 homolog
	c82982	838	PF03762.12	34.07	3.00E-21	Vitelline membrane outer layer protein 1 homolog
	c91269	687	PF03762.12	40	5.00E-15	Vitelline membrane outer layer protein 1 homolog
	c62906	739	PF03762.12	26.74	5.00E-06	Vitelline membrane outer layer protein 1 homolog
	c79584	611	PF03762.12	31.37	8.00E-09	Vitelline membrane outer layer protein 1
	c88421	893	PF03762.12	37.97	8.00E-27	Vitelline membrane outer layer protein 1
	c96540	929	PF03762.12	37.43	5.00E-28	Vitelline membrane outer layer protein 1
	c96540	941	PF03762.12	37.43	5.00E-28	Vitelline membrane outer layer protein 1
	c67628	811	PF03762.12	27.73	7.00E-11	Vitelline membrane outer layer protein 1
3-β-HSD	c79989	1255	PF01073.14	29.48	3.00E-31	3 beta-hydroxysteroid dehydrogenase/Delta 5—>4-isomerase
	c79989	1272	PF01073.14	26.9	2.00E-22	3 beta-hydroxysteroid dehydrogenase/Delta 5—>4-isomerase
MOIH	c97837	1361	PF01147.12	30.71	5.00E-13	Mandibular organ-inhibiting hormone
	c97837	1172	PF01147.12	30.71	5.00E-13	Mandibular organ-inhibiting hormone
	c97837	1467	PF01147.12	30.71	5.00E-13	Mandibular organ-inhibiting hormone
LSHR	c100330	479	PF13855.1	36.43	4.00E-20	Lutropin-choriogonadotropic hormone receptor
	c83529	1329	PF00001.16	56.68	5.00E-84	Lutropin-choriogonadotropic hormone receptor
	c83529	1347	PF00001.16	56.68	6.00E-84	Lutropin-choriogonadotropic hormone receptor
GNRHR	c78057	986	PF00001.16	44.93	1.00E-15	Gonadotropin-releasing hormone receptor
	c78057	1061	PF00001.16	45.59	2.00E-15	Gonadotropin-releasing hormone receptor
	c93526	1895	PF00001.16	38.38	2.00E-34	Gonadotropin-releasing hormone II receptor
	c93526	460	PF00001.16	35.85	1.00E-12	Gonadotropin-releasing hormone II receptor
FSHR	c57473	1429	PF00001.16	46.67	1.00E-55	Follicle-stimulating hormone receptor
	c88521	532	PF00001.16	53.07	2.00E-56	Follicle-stimulating hormone receptor
	c62767	1084	PF13855.1	30.9	2.00E-32	Follicle-stimulating hormone receptor
Estrogen	c98086	1030	PF00685.22	34.62	6.00E-42	Estrogen sulfotransferase
	c96787	2987	PF00685.22	35.15	1.00E-28	Estrogen sulfotransferase
	c96787	1660	PF00685.22	35.15	1.00E-28	Estrogen sulfotransferase
	c51900	1143	PF00105.13	31.54	9.00E-10	Estrogen receptor
	c51900	1284	PF00105.13	31.54	9.00E-10	Estrogen receptor

During the process of oocyte growth, most of the nutrients come from yolk proteins [29], and yolk proteins can be synthesized by the ovaries [30–33]. Our sequencing results showed some fragments of the vitellogenin gene which correspond with results of alternative splicing of vitellogenin [34]. Besides vitellogenin, nine transcripts of vitelline membrane outer layer 1 protein were found in the ovaries (Table 4). The role of this protein in crustaceans is to develop oocytes and avoid mixing of yolk and albumen [35].

The development of crustacean reproductive tissue is a dynamic process involving coordinated interactions between regulators that assemble or edit the cellular constituents that support the developing gametes. Endocrine factors and locally expressed steroids and hormones induce growth, differentiation, and maturation of the follicular cells. The assembling support structures and the maturing follicles both undergo cellular remodeling and organization throughout development. The formation and maturation of the ovary is a very complex process, which requires the cooperation of a spectrum of functional pathways. In the ovary tissue, we found there were 114 putative proteins involved in the pathway of oocyte meiosis, 109 involved in the cell cycle, and 99 involved in ubiquitin-mediated proteolysis (S5 Table).

Besides hormones and yolk-related proteins, other genes were also found in our study. For example, granulin (c86027_g1_i1, S1 Table), which is expressed at high levels in the ovaries and epididymis of rats [36], plays roles in reproductive cell growth and behavior in our crabs. The 95 genes related to lipase and 132 genes related to protease demonstrate that lipids and proteins are also main nutritional sources during ovarian development (S2 Table). Previous studies of *P*. *trituberculatus* ovarian development have also shown higher activity of lipases and proteases [37]. In our results, there were 27 transcripts involved in the biosynthesis of unsaturated fatty acids, but only one involved in saturated fatty acid biosynthesis, which is consistent with the high content of unsaturated fatty acids in crabs [38]. In addition, 68, 60, 25, and 32 transcripts were discovered in the categories of fatty acid metabolism, fatty acid degradation, fatty acid elongation, and fat digestion and absorption, respectively (S2 Table). These pathways may represent a future research direction for the study of lipid metabolism in *P*. *trituberculatus*.

### DEGs in different ovarian development stages

During the exogenous vitellogenic stage of ovarian development, phases III and IV are the begin and peak in *P*. *trituberculatus* [6]. Differential analysis of gene expression between phases III and IV will provide an opportunity to understand the critical genes in the exogenous vitellogenic stage. The aim of our project was to find differentially expressed genes. We identified 36,343 common genes in phase III and IV libraries (S4 Table), of which, 506 (1.39%) were significantly different (Fisher P-test, P<0.05), and 324 (64.03%) of them were annotated successfully.

During the phases III and IV of ovarian development, most genes are expressing continuously, and expression quantity become higher and higher untill the end of ovarian mature. In these DEGs, most genes (over 95%) were upregulated. Of these genes, there were five with little to no expression in phase III but high expression in phase IV: arginine kinase (AK), sodium/glucose cotransporter 4 (SGLT4), 6-O-methylguanine DNA methyltransferase (MGMT), patched (PTCH), and Wnt-4. AK (EC 2.7.3.3) plays an important role in cellular energy metabolism in invertebrates [39, 40] by catalyzing the reversible transfer of a phosphate group from Mg-ATP to arginine. AK belongs to the family of transferases and participates in arginine and proline metabolism. This enzyme is related to protein metabolism which represented that nutrition was provided by protein metabolism during ovarian development. SGLT4 is a member of the glucose transporter family which contributes to glucose reabsorption. SGLT4 proteins use the energy created by the ATPase pump to transport glucose. MGMT is crucial for genome stability. It repairs naturally occurring mutagenic DNA lesions with O-6-methylguanine back to guanine and prevents mismatch and errors during DNA replication and transcription. Accordingly, loss of MGMT increases the carcinogenic risk in mice after exposure to alkylating agents [41]. PTCH is an essential gene in embryogenesis and mutations in this gene may be embryonic lethal. PTCH functions as the receptor for the Hedgehog protein [42] and controls its spatial distribution in part via endocytosis of bound Hedgehog protein [43]. The special gene, however, is Wnt-4, which regulated the female development in mammals. Some previous reports have demonstrated that Wnt-4 is regarded as a sex determination gene for it can form a signaling cascade to inhibit a portion of the testis pathway and allow proper ovarian development to occur. It showed that Wnt-4 played a key role in the morphological development of female mammals [44]. Wnt-4 can also regulate formation of the mullerian duct and generation of ovarian steroids [45] which indicated that Wnt4 may play an important role in ovarian development. Also it needed further confirmation. In the current research, these genes were differentially expressed during stages III and IV of ovaries reflecting their important functions for ovarian development.

Differential analysis of gene expression between phases III and IV showed that predominant gene clusters were found to be involved with various cellular and metabolic biological processes and functions, including the formation of structural components of cells and subcellular organelles. We analyzed the top 100 highly expressed genes which showed in S5 Table. These genes belong to 20 pathway which contained transporters, development, transcription factors, metabolism of other amino acids (Alanine racemase), carbohydrate (probable chitinase 3) and lipid (N-acetylgalactosaminyltransferase 6), solute carrier family members, and enzymes. We found that there are transporters which belong to solute carrier family members such as Solute carrier family 22 member 6-A, Sodium/glucose cotransporter 4, acting during this process. The reproduction and growth of gonads cannot leave the supply of the nutrients. Protein is the first important nutrient for animal growth and reproduction and amino acids are its basic components. Aspartate aminotransferase (AST) can increased protein breakdown to provide the energy requirements [46] and this enzyme was found to exhibit highest activities during the spawning phase [47]. For the development of crab ovarian tissue is dynamic [48], ovarian development is a complex cellular process requiring differential expression of many proteins and phosphorylation/dephosphorylation of several key proteins [49, 50]. In the transcriptomic approach, only those showing differential expression are considered. Accordingly, typical proteomics and phosphoproteomics should be further carried out to complete the knowledge on ovarian development of this important species. The current study furthers our understanding of the molecular and physiological nature of the crab ovary, which will provide insight for aquaculture practice.

## Conclusions

This study is the first time to investigate the refined process of ovary development in *P*. *trituberculatus* by 454 transcriptome sequencing. Our transcriptome sequencing produced 126,075 contigs for the swimming crab. Further analysis revealed 36,343 contigs with ORFs were found in the ovarian development process. DEGs analysis revealed that 506 significant genes were found between phases III and IV of ovarian development. Although we have only recently begun to study reproductive regulatory mechanisms at a molecular level in *P*. *trituberculatus*, the knowledge gained from these studies is proving insightful information. In future studies we will focus on the important signaling pathways especially with respect to some factors that are associated with fertilization. We can use these bioinformation to evaluate the nutrient status of *P*. *trituberculatus*, also can propose better feeding pattern to improve the quality of *P*. *trituberculatus* with these information. Our results not only provided more transcriptome information for the understanding of ovarian development in *P*. *trituberculatus*, but also were helpful to further investigations of functional genomics for this species. Overall, the current study provided more information for us to understand the molecular process of the ovarian development, which will provide more useful insight for aquaculture practice.

## Supporting Information

S1 Fig Correlation of gene expression. Heatmap shows the Pearson correlation between gene expression of 4 libraries (RQ12BJ00204 and RQ12BJ00206 from phase III, RQ12BJ00210 and RQ12BJ00212 from phase IV).
(TIF)

S1 Table Annotation results of predicted ORF. (XLSX)

S2 Table The number of genes assigned to KEGG pathways. (XLSX)

S3 Table Summary of single-end RNA sequencing results from *P*. *trituberculatus* ovaries in phase III and phage IV. (XLSX)

S4 Table DEGs between phase III and phase IV. (XLSX)

S5 Table Top 100 genes which upregulated in phase IV. (XLS)
