# Supplementary figures and images for: Ovarian Transcriptome Analysis of Portunus trituberculatus Provides Insights into Genes Expressed during Phase III and IV Development
Source: PLoS One. 2015 Oct 2;10(10):e0138862. doi: 10.1371/journal.pone.0138862 (PMC4591999; doi:10.1371/journal.pone.0138862)

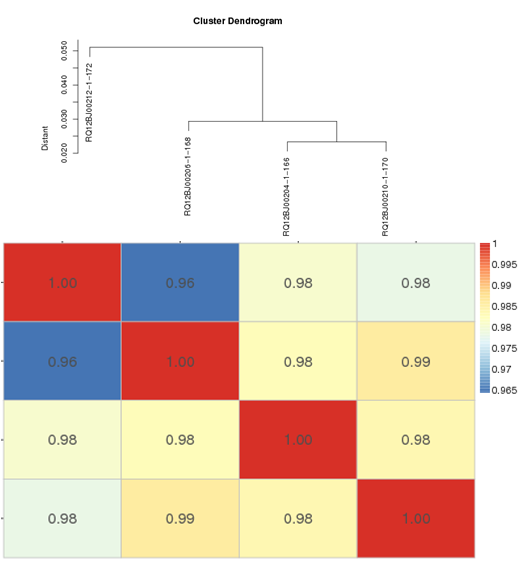

Supplement: S1 Fig — Heatmap shows the Pearson correlation between gene expression of 4 libraries (RQ12BJ00204 and RQ12BJ00206 from phase III, RQ12BJ00210 and RQ12BJ00212 from phase IV). (TIF) [file pone.0138862.s001.tif]
